# Targeting Glycans and Heavily Glycosylated Proteins for Tumor Imaging

**DOI:** 10.3390/cancers12123870

**Published:** 2020-12-21

**Authors:** Ruben D. Houvast, Mireille Vankemmelbeke, Lindy G. Durrant, Manfred Wuhrer, Victor M. Baart, Peter J. K. Kuppen, Lioe-Fee de Geus-Oei, Alexander L. Vahrmeijer, Cornelis F. M. Sier

**Affiliations:** 1 Department of Surgery, Leiden University Medical Center, 2333 ZA Leiden, The Netherlands; R.D.Houvast@lumc.nl (R.D.H.); V.M.baart@lumc.nl (V.M.B.); P.J.K.Kuppen@lumc.nl (P.J.K.K.); A.L.vahrmeijer@lumc.nl (A.L.V.); 2 Scancell Limited, University of Nottingham Biodiscovery Institute, University Park, Nottingham NG7 2RD, UK; MireilleVankemmelbeke@scancell.co.uk (M.V.); Lindy.Durrant@nottingham.ac.uk (L.G.D.); 3 Division of Cancer and Stem Cells, School of Medicine, University of Nottingham Biodiscovery Institute, University Park, Nottingham NG7 2RD, UK; 4 Center for Proteomics and Metabolomics, Leiden University Medical Center, 2333 ZA Leiden, The Netherlands; m.wuhrer@lumc.nl; 5 Department of Radiology, Section of Nuclear Medicine, Leiden University Medical Center, 2333 ZA Leiden, The Netherlands; l.f.de_geus-oei@lumc.nl; 6 Biomedical Photonic Imaging Group, University of Twente, 7500 AE Enschede, The Netherlands; 7 Percuros BV, 2333 ZA Leiden, The Netherlands

**Keywords:** cancer, aberrant glycosylation, carbohydrates, gangliosides, mucins, proteoglycans, molecular imaging, biomarkers

## Abstract

**Simple Summary:**

Distinguishing malignancy from healthy tissue is essential for oncologic surgery. Targeted imaging during an operation aids the surgeon to operate better. The present tracers for detecting cancer are directed against proteins that are overexpressed on the membrane of tumor cells. This review evaluates the use of tumor-associated sugar molecules as an alternative for proteins to image cancer tissue. These sugar molecules are present as glycans on glycosylated membrane proteins and glycolipids. Due to their location and large numbers per cell, these sugar molecules might be better targets for tumor imaging than proteins.

**Abstract:**

Real-time tumor imaging techniques are increasingly used in oncological surgery, but still need to be supplemented with novel targeted tracers, providing specific tumor tissue detection based on intra-tumoral processes or protein expression. To maximize tumor/non-tumor contrast, targets should be highly and homogenously expressed on tumor tissue only, preferably from the earliest developmental stage onward. Unfortunately, most evaluated tumor-associated proteins appear not to meet all of these criteria. Thus, the quest for ideal targets continues. Aberrant glycosylation of proteins and lipids is a fundamental hallmark of almost all cancer types and contributes to tumor progression. Additionally, overexpression of glycoproteins that carry aberrant glycans, such as mucins and proteoglycans, is observed. Selected tumor-associated glyco-antigens are abundantly expressed and could, thus, be ideal candidates for targeted tumor imaging. Nevertheless, glycan-based tumor imaging is still in its infancy. In this review, we highlight the potential of glycans, and heavily glycosylated proteoglycans and mucins as targets for multimodal tumor imaging by discussing the preclinical and clinical accomplishments within this field. Additionally, we describe the major advantages and limitations of targeting glycans compared to cancer-associated proteins. Lastly, by providing a brief overview of the most attractive tumor-associated glycans and glycosylated proteins in association with their respective tumor types, we set out the way for implementing glycan-based imaging in a clinical practice.

## 1. Introduction

Cancer is a leading cause of death worldwide, accompanied by a high burden on society. Biomedical imaging of malignant tissue plays a pivotal role in cancer detection, biopsy/therapeutic guidance, and monitoring, and, thus, is a major contributor in defining treatment and surgical planning [1]. Current imaging methodologies such as X-ray, ultrasound (US) computed tomography (CT), (functional) magnetic resonance imaging ((f)MRI), positron emission tomography (PET), and single-photon emission computed tomography (SPECT) are routinely applied within the standard of care before surgery takes place [1,2]. Untargeted techniques, such as X-ray, US, and CT, detect tissue irregularities based on anatomy and are, therefore, not exclusively specific for neoplastic tissue. Since tumor-targeted contrast agents provide a more specific indication of molecular processes in both premalignant lesions and tumors, their employment is of particular interest for preoperative staging, intraoperative detection, and postoperative monitoring of cancer.

An adequate tumor-to-background ratio (TBR), which allows a clear differentiation between healthy and malignant tissue, is the cornerstone of tumor imaging [3]. To maximize the TBR, an imaging target should be highly and homogenously expressed, ideally confined to tumor tissue only. Since the most available protein-based imaging targets appear to have limitations, such as substantial expression on normal surrounding tissues or lack of overexpression in early disease stages, the search for novel targets is an ever-continuing topic of research.

Aberrant glycosylation represents a hallmark of cancer, offering a set of novel tumor-specific targets [4]. In man, more than half of all membrane-bound or soluble, secreted proteins carry sugar molecules, referred to as glycans. These proteins are, therefore, categorized as glycosylated proteins or, in short, glycoproteins. Glycans can also be attached to lipids, forming glycolipid structures, such as gangliosides [5,6]. Of note, particular glycoproteins, such as proteoglycans and mucins, carry an extensive amount of glycans that account for the majority of their molecular weight and size, while extensively orchestrating their function. These glycoproteins are further referred to as heavily glycosylated proteins.

In cancer and other pathological process, including infection and chronic inflammation, glycans and heavily glycosylated proteins, which are intricately linked to disease progression, become overexpressed [7,8,9,10]. Despite the tumor-specific expression of these structures, only a few of these determinants have, so far, been validated as targets for tumor imaging. Table 1 summarizes the recent studies evaluating tumor-associated glycans and heavily glycosylated proteins as targets for molecular imaging of cancer and provides an overview of the most promising targets with respect to their tumor type. In this review, we provide a background on the most promising glycome targets and highlight the great potential of these structures as imaging targets by discussing the recent preclinical and clinical research into glycan-related tumor imaging.

## 2. Glycans

### 2.1. Background

**Figure 1:**
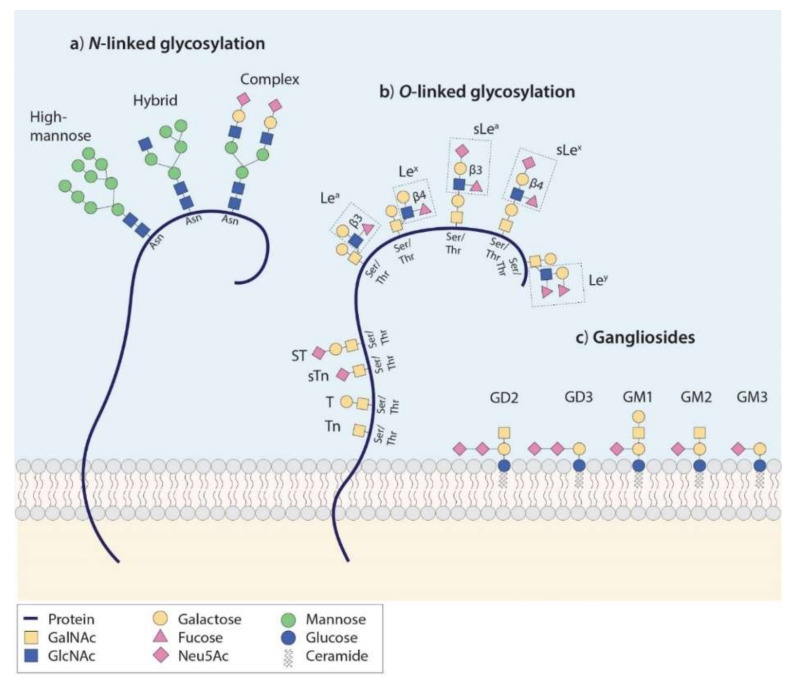
Schematic structures of oligosaccharides: (**a**) N-Linked glycans are covalently attached to proteins via asparagine (Asn). *N*-Glycans are assigned to three groups in which all share the same Pentasaccharide, Trimannosyl core structure: (1) High-Mannose *N*-glycans (2) Hybrid *N*-glycans in which the core is extended via both mannose and *N*-Acetylglucosamine (GlcNAc) residues and (3) complex *N*-glycans in which GlcNAc-initiated antennae are present. (**b**) *O*-linked glycans are covalently attached to proteins via Serine (Ser) or Threonine (Thr). Mucin-type *O*-Glycans are initiated by *N*-Acetylgalactosamine (GalNAc), while elongated, GlcNAc-containing glycans (displayed in dashed boxes) contribute to Type 1 (Galβ1, 3GlcNAc) and Type 2 (Galβ1, 4GlcNAc) structures. In this figure, sLe^a^ and sLe^x^ extend from a core 1 structure (Galβ1–3GalNAc), while Le^a^, Le^x^, and Le^y^ are attached to a core 2 structure (Galβ1, 3[β1, 6lcNAc]GalNAc). Both *N-*and *O-*glycan antigens can carry *N*-Acetylneuraminic (Neu5Ac) acids, categorizing these structures as sialylated antigens. (**c**) Gangliosides consist of varying arrangement of sialic acid-containing glycan chain attached to the cell membrane via a lipid anchor, the ceramide. GM1 to GM3 are initiated by glucose and carry one sialic acid, while GD2 and GD3 carry two sialic acids.

The attachment of glycans to proteins occurs mainly in two forms, namely *O*-and *N*-linked. *O*-linked glycosylation occurs via the attachment of a sugar molecule to the hydroxyl group of mainly serine (Ser) or threonine (Thr) residue side chains in a protein, whereas *N*-linked glycosylation occurs via the attachment of an oligosaccharide consisting of multiple sugar molecules to the nitrogen atom of asparagine (Asn) side chains (Figure 1a,b) [78]. *N-*glycans, which all share a common glycan core, can be grouped into high-mannose, hybrid, and complex *N-*glycan structures, as depicted in Figure 1a. However, since the development of *N-*glycan-specific targeting vehicles is challenging due to the extensive structural similarity of *N-*glycans, therapeutic and imaging tracer development generally focusses on *O*-linked glycans (explained in detail in Section 6: Targeting the Glycome: Opportunities and Challenges).

The most abundant form of *O-*glycosylation is mucin-type (GalNAc) *O*-glycosylation in which extracellular or secreted glycoproteins are modified with *N*-acetylgalactosamine residues (GalNAc-alpha-*O*-Ser/Thr) that, by the addition of Galactose and *N*-acetylglucosamine (GlcNAc) residues, constitute different *O*-GalNAc core structures. *O-*glycan cores can be further elongated by the addition of additional monosaccharides, which results in specific terminal glycan motifs, of which some are shown in Figure 1b. These structures play roles in biological processes such as cell adhesion, receptor activation, cell growth, signal transduction, apoptosis, and endocytosis and may confer antigenicity or provide cell protection by contributing to the glycocalyx formation [79,80,81].

### 2.2. Aberrant Glycosylation in Cancer

In cancer, aberrant glycosylation is mainly characterized by increased *N*-glycan branching, augmented *O*-glycan density, incomplete glycan synthesis, and, in more advanced cancers, synthesis of neo-glycan determinants that carry large amounts of sialic acids or fucose residues [10,11,12,13,14,15,16,17,18,19,20,21,22,23,24,25,26,27,28,29,30,31,32,33,34,35,36,37,38,39,40,41,42,43,44,45,46,47,48,49,50,51,52,53,54,55,56,57,58,59,60,61,62,63,64,65,66,67,68,69,70,71,72,73,74,75,76,77,78,79,80,81,82]. This leads to the appearance of immature truncated GalNAc/mucin-type *O*-glycans, such as sialyl-Thomsen-nouveau (sTn) and complex versions of Lewis glycans, such as sialyl-di-Lewis^a^ (s-di-Le^a^) [83]. In addition, overexpression of normally expressed Lewis glycan antigens, such as sialyl-Lewis^a^ (sLe^a^, known as CA19-9) and its structural isomer sialyl-Lewis^x^ (sLe^x^) is observed. sTn, its non-sialylated counterpart Tn, and Lewis glycans are extensively expressed in a wide variety of epithelial-derived cancers [9,84] such as cancers of the digestive tract [8,85,86,87], breast [86,88,89,90], lung [86,91,92], bladder [86,90,93], and ovaries [86,87,94]. Figure 1b depicts the schematic structure of these and other tumor-associated *O*-glycoantigens and illustrates the most frequently observed *O*-GalNAc core structures from which they extend.

Tumor-associated glycans are heavily involved in tumor progression both directly or indirectly by influencing its protein or lipid carrier’s function [95]. For instance, both sLe^x^ and sLe^a^ can serve as ligands for E-selectins and P-selectins present on endothelial cells, thereby facilitating cell adhesion, extravasation, and metastasis [85]. sLe^a^ is overexpressed on a wide variety of tumor-associated glycoproteins, including mucin-1 (MUC1), MUC5AC, and MUC16 (CA125) [96,97]. Moreover, sLe^x^, also called CD15s, is overexpressed on liver acute-phase proteins, including haptoglobin [98] and ceruloplasmin [99] and on mucins MUC1, MUC5AC, and MUC6 in pancreatic cancer [97,98,99,100]. This suggests a major advantage of targeting glycans in relation to protein targeting, as multiple tumor-associated proteins can be targeted simultaneously via a single glycan motif (described in detail in Section 6: Targeting the Glycome: Opportunities and Challenges). Moreover, glycans are, in relation to proteins, very densely distributed on the outermost layer of the cell membrane [101], making them easily accessible for targeting vehicles and, consequently, attractive targets for imaging.

### 2.3. Imaging of sTn

Despite its abundant expression in a wide variety of carcinomas, e.g., lung, ovarian, bladder, breast, and almost all gastrointestinal cancers with low normal tissue distribution (reviewed in Reference [84]). Studies into sTn-targeted molecular imaging have particularly focused on colorectal tumors and reported clear tumor delineation. sTn, which is overexpressed on mucins MUC1 [102,103], MUC2 [97], MUC5AC [97], and MUC6 [97], and oncoprotein CD44(v6) [103,104], has been evaluated as a target in several imaging studies aiming to optimize a SPECT imaging contrast in colorectal cancer. For instance, Rossin et al. described a strategy using anti-sTn monoclonal antibody (mAb) CC49 to pre-target tumor cells prior to administration of two mAb-clearing agents followed by administration of the CC49-binding radiolabel. Pre-targeting could theoretically provide higher tumor-to-background contrast by clearing unbound CC49 mAbs from the circulation [20]. LS174T colon carcinoma-carrying mice that were administered with clearing agents showed a remarkable 125-fold improvement of the tumor-to-blood ratio 3 h post-injection, when compared to administration of (non-pre-targeted) ^177^Lu-NOTA-CC49.

Apart from SPECT imaging, sTn has been evaluated as a target for real-time intra-operative imaging using near-infrared fluorescent (NIRF) light. For instance, murine CC49 and its humanized, CH2-deleted variant HuCC49ΔC_H_2, were conjugated to NIR dye Cy7 and evaluated for NIRF imaging in a subcutaneous mouse model of colorectal cancer [16]. Administration of murine CC49–Cy7 allowed clear tumor visualization with a tumor-to-blood ratio of 15.5 at 96 h post-injection. Even though its humanized counterpart was cleared roughly twice as fast, it showed a tumor-to-blood ratio of 12.0 at 18 h post-injection with a low specific uptake in other organs, confirming the great potential of sTn as a target for imaging of colorectal tumors. However, since Cy7 has not been clinically approved, the translational potential of the CC49–Cy7 tracer in this confirmation is currently limited. Apart from NIRF imaging, the same research group conjugated HuCC49ΔC_H_2 to ^124^I and showed excellent tumor delineation using PET imaging in the same mouse model [17].

The low immunogenicity of glycans has both challenged therapeutic efficacy as well as the development of specific and high-affinity IgG mAbs (described in detail in Section 6: Targeting the Glycome: Opportunities and Challenges) [105,106]. For instance, sTn antibodies have been shown to additionally react with the non-sialylated Tn epitope and binding to sTn that was dependent on the glycoprotein it was expressed on. Thus, the need for novel antibodies that could serve as a sTn-targeting moiety arose [86,107]. For example, Loureiro et al. developed and characterized the novel sTn mAb L2A5, which showed tumor-specific reactivity with all included bladder and colorectal cancer tissues, and 20% of triple-negative breast cancer tissues [90]. Moreover, Prendergast et al. developed a panel of murine, high-affinity, internalizing sTn antibodies that showed positive immunohistochemical staining of the majority of human ovarian, bladder, colorectal, pancreatic, lung, and gastric tumor tissues, with low reactivity to normal human tissues [86]. Two mAbs, 5G2-1B3 and 2G12-2B2, were subsequently humanized, whilst maintaining limited reactivity with normal human tissues. Of these mAbs, 2G12-2B2-MMAE showed effective tumor targeting by inhibiting tumor growth in both an ovarian cancer cell line and patient-derived ovarian xenograft mouse models [87]. Thus, sTn, although underexplored, may pose a very suitable target considering its potential employment for imaging of a wide range of tumor types [84].

### 2.4. Imaging of sLe^a^/CA19-9

Considering its clinically applied tumor-specific upregulation in tissues and sera of pancreatic cancer patients, sLe^a^/CA19-9 has been exploited as a target for imaging and therapy [23,25,26,28,29,30,31]. Houghton et al. have used the humanized mAb 5B1 conjugated to a NIRF dye and radionuclide ^89^Zr [108] for bimodal fluorescence/PET imaging of pancreatic tumors, resulting in an excellent tumor, a positive lymph node, and metastases localization in both a subcutaneous and orthotopic metastasizing mouse model [31]. To improve tumor/non-tumor contrast even further, several studies using anti-sLe^a^ mAbs to pre-target tumor cells before radiolabel administration have been developed, which have led to remarkable improvements in TBR via various techniques [28,29]. 

During malignant transformation, sLe^a^ but also sLe^x^, become expressed on the glycoprotein CEA (carcinoembryonic antigen) [109,110], which is currently undergoing clinical translation facilitated by our group as a target for NIRF imaging of colorectal cancer (SGM-101, SurgiMab) [111]. Given their wide expression across different tumor types and multiple tumor-associated proteins, sLe^a^ and sLe^x^, may be very suitable candidates for pan-carcinoma tumor imaging. 

Several mAbs recognizing alternative Lewis glycans have been developed and described. Noble et al. described the mAb 692/29, which recognizes a unique set of Lewis^y^ and Lewis^b^ glyco-epitopes. Despite some reactivity with normal gastrointestinal epithelia, 696/29, bound to 82% of colorectal tumors showed inhibition of cell growth in vivo that was further supplemented by chemotherapy [112]. More recently, the novel murine/chimeric IgG mAbs FG88.2 and FG129/CH129 were described, which were bound to Lewis^a/c/x^-related and sialyl-di-Lewis^a^ glyco-epitopes, respectively, that were highly expressed on pancreatic, colorectal, stomach, lung, and ovarian carcinomas with restricted expression in normal tissues [83,113]. Subsequently, our group evaluated IRDye 800CW-conjugated FG88.2 and its chimeric mouse/human counterpart, CH88.2, for real-time NIRF imaging in subcutaneous mouse models of colon and pancreatic cancer, which provided excellent tumor localization and delineation using a clinical camera system [22].

Lastly, Shimomura et al. described an alternative approach for glycan-targeting by using glycan-binding lectin rBC2LC-N, which binds to type 1 (Fucα1-2 Galβ1-3GlcNAc) and type 3/4 fucosylated glycans (Fucα1-2 Galβ1-3GalNAc, see Figure 1a). This showed reactivity with almost all tested human pancreatic ductal adenocarcinoma (PDAC) specimens [114]. After conjugation with a bacterial exotoxin, a remarkable cytotoxicity was observed in several patient-derived models, suggesting excellent tumor penetration. Most importantly, the authors excluded that rBC2LC-N caused human serum agglutination in vitro, which is a frequently observed phenomenon after lectin administration [115]. These results not only pave the way for a potential imaging strategy for PDAC, but also provide a novel explorable approach for glycan targeting. 

### 2.5. Glycan Imaging in the Clinic

Several glycan-targeting imaging tracers have already been evaluated in a clinical setting. For instance, anti-sTn mAb CC49 and its predecessor mAb B72.3 were conjugated to ^125^I and used as a tracer for radio-immuno-guided surgery (RIGS) of colorectal cancer [64,65,66,67,68]. Intraoperatively, RIGS using ^125^I-CC49 allowed detection of 86% and 97% of primary and recurrent tumors, respectively, while the surgical resection was influenced in roughly half of the cases [65]. However, routine clinical implementation of RIGS is limited by the inconveniently long period between tracer administration and surgery (one week) and handling of the long-lived ^125^I isotope, and, therefore, RIGS has been overtaken by other imaging strategies, such as NIRF imaging [116]. In addition, the anti-CA19.9 tracer ^89^Zr-DFO-HuMab-5B1 (MVT-2163) has recently been evaluated in a Phase I trial for PET imaging of pancreatic cancer and allowed high-contrast imaging of tumors and metastases, including lesions that were not detected with traditional imaging methods [69]. Administration of MVT-2163 was safe, causing mild to moderate side effects on the first day, including nausea, fever, and hypertension, in 50% of patients.

## 3. Gangliosides

### 3.1. Background

Gangliosides are sialic acid-containing glycosphingolipids (a glycolipid subclass) that are attached to the cell membrane via a lipid portion: the ceramide (Figure 1c). These structures are abundantly present in the central nervous system where they serve pivotal roles in its development and maintenance [117]. Simple ganglioside structures, such as GD2, GD3, or GM1-3, are aberrantly expressed in several neuroectodermal-derived cancers, including melanoma, osteosarcoma, and neuroblastoma, and in breast cancer [118,119,120,121]. Several studies have shown that gangliosides are involved in tumor cell proliferation, mobilization, and metastasis [122,123].

In 2015, the human/mouse chimeric anti-GD2 mAb ch14.18 (dinutuximab) became the first and only FDA-approved therapeutic anti-glycan antibody. Administered in combination with IL-2, GM-CSF, and isotretinoin, dinutuximab increased by two-year event-free, and overall survival (OS) rates of high-risk neuroblastoma patients with 20% and 10.9%, respectively [124,125]. Phase II and III dinutuximab trials in neuroblastoma (NCT02743429, NCT01704716), osteosarcoma (NCT02484443), and small cell lung cancer (NCT03098030) are underway.

### 3.2. Imaging of GD2

Several studies have validated the humanized variant of dinutuximab, hu14.18, as a targeting moiety for tumor imaging. For instance, Vāvere et al. validated the hu14.18K322A variant of hu14.18, which was developed to decrease neuropathic pain after administration while maintaining cytotoxicity, as a targeting moiety for PET imaging. Administration of ^64^Cu-labeled hu14.18K322A to GD2-postive M21 melanoma xenograft-carrying mice allowed excellent tumor delineation and localization with low tracer uptake in other organs [36]. More recently, the same group additionally validated the tracer in a patient-derived and metastatic orthotopic in vivo model of osteosarcoma and observed similar tumor-specificity related to GD2 expression and detected tumor lesions as small as 29 mm^3^ at 48 h after injection [37]. Dinutuximab-beta (ch18.18/CHO), which is an FDA-approved biosimilar variant of ch14.18, was recently conjugated to IRDye 800CW and evaluated for NIRF imaging of neuroblastoma in a mouse model [35]. At four days post-injection, the tracer showed high-contrast tumor accumulation in both orthotopic transplanted human KCNR neuroblastoma cells and patient-derived organoid xenograft mouse models. Moreover, the authors showed that neoadjuvant anti-GD2 immunotherapy did not influence tracer uptake, supporting an application of the tracer in a clinical setting. Alternatively, Jiao et al. used gold nanoparticles (GNPs) conjugated to hu14.18K322A as NK-cell activators as well as CT contrast agents [126]. After incubating NB1691 neuroblastoma cells and M21 melanoma cells with the construct, the authors observed a two-fold higher antibody-dependent cellular cytotoxicity efficacy, along with a 5-fold to 8-fold increase in CT imaging contrast compared to controls, proposing a potent bimodal application of the tracer. None of the previously mentioned studies evaluated neurotoxicity following administration of anti-GD2 contrast agents’ administration. Providing that imaging tracers are administrated in a substantially lower dose than therapeutic agents, one may expect that neurotoxicity will not pose a limiting factor for GD2-based imaging.

### 3.3. Ganglioside-Based Nerve Imaging

Although other gangliosides have been established as not specific for tumor cells, they might still be of use as targets for imaging during oncological surgery. For instance, to avoid nerve injury during prostatectomy, surgeons may be assisted by a real-time nerve monitoring system, based on a ganglioside specific NIRF tracer. Massaad et al. used the anti-GT1b-2b mAb, which has been shown to bind axons in spinal roots, peripheral nerves, and neurons of dorsal root ganglia and the spinal cord, as a targeting moiety [127]. Conjugated to fluorescent dye Dylight550, peripheral nerves could be imaged using the tracer from 24 hours up to 20 days after intravenous administration to wild-type mice. Furthermore, the authors reported that GT1b-2b-induced nerve fiber damage was not present.

## 4. Proteoglycans

### 4.1. Background

**Figure 2:**
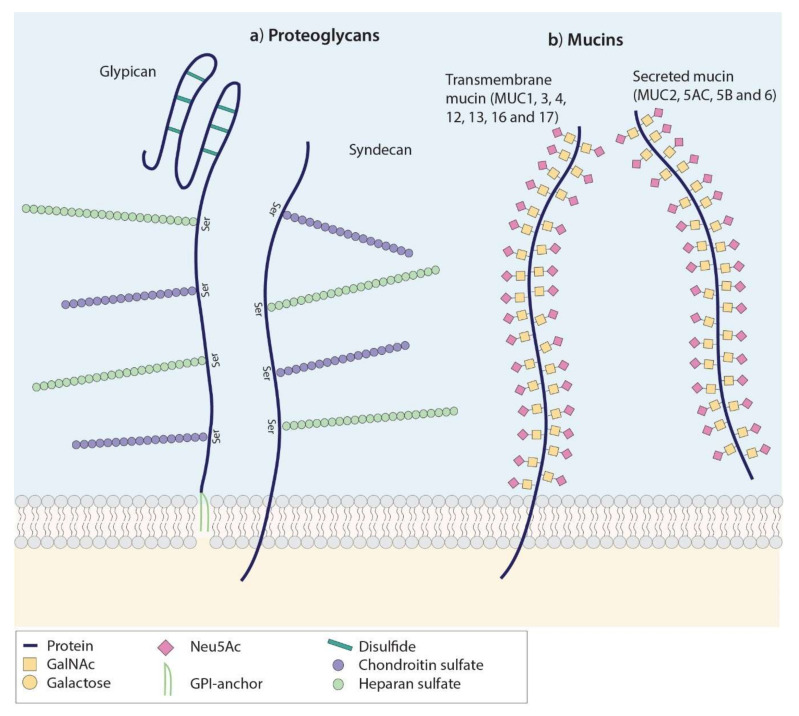
Schematic structure of heavily glycosylated glycoproteins. (**a**) Proteoglycans are transmembrane glycoproteins that consist of a core protein decorated with glycosaminoglycan (GAG) chains. In glypicans, the protein core is stabilized by disulphide bridges and linked to the cell membrane via GPI-anchors. Both glypicans and syndecans contain serine-linked heparin sulphate and chondroitin sulphate GAGs at both sides of the protein (here only depicted on one side), classifying them as HSPGs. (**b**) Mucins are high-molecular weight proteins that are extensively decorated with mucin-type *O-*Glycans, schematically illustrated here by the sTn epitope. Mucins are subdivided into transmembrane (MUC1, MUC3, MUC4, MUC12, MUC13, MUC16, and MUC17) and secreted mucins (MUC2, MUC5AC, MUC5B, and MUC6).

Heavily glycosylated proteins, such as proteoglycans, also form an interesting array of targets for tumor imaging, in addition to tumor-associated glycans Figure 2a. Proteoglycans (PGs) consist of linear polysaccharide chains (glycosaminoglycans, GAGs) that are covalently attached to a protein core. PGs form a major component of the extracellular matrix and contribute significantly to the structural integrity of tissues [128]. Moreover, PGs play multifaceted roles in the regulation of essential signaling pathways that are involved in cell proliferation, adhesion and migration, apoptosis, and angiogenesis [129]. Heparan sulfate proteoglycans (HSPGs), such as syndecans and glypicans, have gained significant scientific interest within the oncological field [130,131]. Syndecans and glypicans are localized at the cell surface, allowing them to be heavily involved in integrin and growth factor signaling and regulation of Wnt and Hedgehog signaling, which are pathways known to be dysregulated in cancer [132,133,134,135]. Many recent studies reported overexpression [131] and, understandably, great involvement of HSPGs in carcinogenesis and tumor progression in a wide range of tumors, making these structures potential targets for molecular imaging of cancer [129,131,135,136,137,138].

### 4.2. Imaging of Syndecan-1

Syndecan-1 (CD138) was evaluated as a target for bimodal NIRF imaging and multispectral optoacoustic tomography (MSOT, also known as photoacoustic imaging) in an orthotopic in vivo model of pancreatic cancer [38]. At 6 h post-injection, the fluorescent tumor signal was undetectable, while MSOT provided a clear high-contrast imaging of tumor location with inferior liver and kidney uptake. Taken together, these results underline both the advantage of MSOT imaging in relation to NIRF imaging, i.e., deeper imaging depth as well as the great potential of this syndecan-1 tracer for combined MSOT/NIR imaging of a wide arrange of tumors, given the broad tumor expression of syndecan-1 [138].

More recently, Bailly et al. compared the mAb-based syndecan-1 tracer ^64^Cu-TE2A-9E7.4 with the conventional tracer ^18^F-FDG and ^64^CuCl_2_ for PET imaging of primary multiple myeloma lesions and metastases using a syngeneic mouse model [39]. Although ^64^Cu-TE2A-9E7.4 was found to accumulate in the liver, spleen, kidneys, and digestive tract, the tracer outperformed both ^18^F-FDG and ^64^CuCl_2_ in terms of non-tumor uptake and tumor-to-blood contrast (41 at 24 h post-injection). Moreover, the tracer allowed high-contrast imaging of most metastatic depositions of which one was not observed using ^18^F-FDG.

### 4.3. Imaging of Glypican-3

Glypican-3 is a highly sensitive biomarker of hepatocellular carcinoma (HCC) [139,140]. Over 70% of HCC cases express high levels of glypican-3, while expression is absent on benign hepatic lesions and normal liver tissue [136,140,141]. A vast number of studies evaluated glypican-3 as a PET, ultrasound (US), NIR, and MR imaging target for HCC using several targeting moieties and conjugates of a different origin [40,41,42,43,44,142]. In all in vivo studies, HepG2 cells could be clearly localized and delineated using a glypican-3-specific tracer. Meanwhile, glypican-3 is validated as a target for immunotherapy of HCC in various phase I trials, paving the way for a potential combined therapeutic/imaging application [143].

## 5. Mucins

### 5.1. Background

Mucins form another class of high molecular weight proteins heavily glycosylated with truncated *O*-glycans (Figure 2b) [144]. These often negatively charged sugar branches on both transmembrane (MUC1, MUC4, MUC13, and MUC16) and secreted mucins (MUC2, MUC5AC, MUC5B, and MUC6) form a physical barrier, protecting the underlying epithelium [144,145,146]. In cancer, aberrantly glycosylated mucins become overexpressed and are, directly or indirectly via their truncated sTn/Tn/TF (Thomsen-Friedenreich) glyco-epitopes, heavily involved in proliferation, migration, invasion, metastasis, and chemo-resistance, and radio-resistance of tumor cells [147,148,149,150,151,152]. For instance, both MUC1, also called epithelial membrane antigen (EMA), and MUC16, also called CA125, are overexpressed in a wide variety of cancer types, including breast [153], lung [154,155], gastrointestinal [146,151,156,157], head-and-neck [158,159], ovarian [160,161], and other gynecological malignancies [162,163], making them potential targets for pan-carcinoma imaging.

### 5.2. Imaging of MUC1

Several preclinical studies described MUC1 as a promising target for molecular imaging [45,46,47,48,49,50,51,52,53,55]. For instance, Chen et al. evaluated MUC1-specific aptamers, conjugated to indocyanine green (ICG) as a fluorescence imaging tracer in breast, non-small cell lung, or hepatocellular carcinoma-bearing mice [52]. The tracer showed fast clearance via the kidneys, while still providing tumor-to-background ratios of 4.0 ± 0.2 in low MUC1-expressing HepG2 tumor cells. Tumors could be clearly localized and delineated in all models.

The expression of MUC1 on the apical surface of normal glandular epithelial cells may reduce tumor-to-background contrast, thus, limiting the application of MUC1-targeting contrast agents [47]. Although most mAbs recognize MUC1 irrespective of its glycosylation pattern, several targeting moieties target a highly tumor-specific conformational MUC1 epitope induced through increased expression of truncated *O-*glycans sTn and Tn. The under-glycosylated (u) MUC1 or (tumor-associated) TA-MUC1 epitope, which becomes expressed on the entire cell surface. Zhao et al. described a promising alternative for serum marker-based therapeutic response monitoring by developing the bimodal MR/fluorescence imaging probe MN-EPPT, which targets uMUC1 [45,46,47,48]. Using spontaneous, human uMUC1-expressing mouse models of breast and pancreatic cancer, uMUC1 expression was detected using MR and fluorescence imaging as early as ductal carcinoma in situ (DCIS) and pancreatic intraepithelial neoplasia (PanIN) lesions onward [46,47]. Tracer uptake decreased after treatment with chemotherapy, suggesting a decrease of uMUC1 expression [45,46,47]. Conversely, increased tracer uptake after chemotherapy was observed in unresponsive tumors, even before anatomical changes were present, indicating uMUC1 as a marker for in vivo imaging of in situ lesions and chemoresistance and/or tumor progression [45,47]. Meanwhile, positive TA-MUC1 expression has been shown in non-small cell lung, ovarian, breast, gastric, colorectal, liver, cervical, kidney, thyroid, and other (non-epithelial) cancers [164,165]. Administration of gatipotuzumab (previously known as PankoMab-GEX), a humanized mAb that binds TA-MUC1 in a Tn/TF-dependent manner, was found safe and was well-tolerated in patients with advanced carcinomas, suggesting a potential pan-carcinoma imaging application of the targeting moiety [166].

Alternatively, GGSK-1/30, which is a murine mAb specific for an alternative MUC1 glycoprotein epitope, was conjugated to ^89^Zr and evaluated for combined PET/MRI imaging of breast cancer-bearing human MUC1-expressing transgenic mice [54]. At 72 h post-injection, administration of ^89^Zr-GGSK-1/30 revealed high tracer tumor uptake with lower uptake in excreting organs and healthy mammary tissue, providing high-contrast tumor delineation. Considering its expression in 90% of breast tumors, including triple-negative breast carcinomas, GGSK-1/30 also seems a promising targeting moiety for pan-breast cancer detection [54].

### 5.3. Imaging of MUC1/MUC5AC:PAM4-Based Systems

The PAM4 mAb, which recognizes a carbohydrate-induced conformational epitope on MUC1 and MUC5AC, has been evaluated as a targeting vehicle for therapy and imaging of pancreatic cancer. PAM4 stained approximately 85% of pancreatic carcinomas, while reactivity with pancreatitis and healthy pancreatic tissue was, respectively, less than 25% and absent [167]. Moreover, the PAM4-epitope is abundantly expressed in PDAC precursor lesions, namely in intraductal papillary mucinous neoplasms and from earliest PanIN lesions (PanIN-1A) onward, suggesting a role for PAM4 in early pancreatic cancer detection [168].

Several studies evaluated PAM4-based contrast agents for γ-scintigraphy of PDAC. Cardillo et al. used bsPAM4 to pre-target Capan-1 pancreatic tumor cells, following administration of histamine-succinyl-glycine peptide haptens ^111^In-IMP-156 or ^99m^Tc-IMP-192, that were developed to interact with bsPAM4 [58]. Subcutaneous Capan-1 xenografts could be imaged as early as 0.5 h after peptide hapten administration. At 3 h post-injection, high tumor-to-blood ratios of 36.5 ± 8.3 and 9.9 ± 5.2 were achieved using ^111^In-IMP-156 and ^99m^Tc-IMP-192, respectively, which was significantly higher when compared to administration of direct-labeled bsPAM4 F(ab’)_2_. More recently, the same group developed the bispecific, trivalent mAb TF10, which consists of two PAM4-derived Fab’ fragments and one mAb-679-derived Fab’, enabling interaction with the radio-labeled hapten-peptide ^111^In-IMP-288 [57]. At 3 h post-injection to Capan-1 tumor-bearing mice, a tumor-to-blood ratio of 915.2 ± 404.3 was observed (vs. 5.2 ± 1.0 using ^111^In-DOTA-PAM4 IgG at 24 h), allowing clear delineation of small tumor lesions. These results clearly show the advantages of a pre-targeting regime that both demonstrate the feasibility of PAM4-based systems for molecular imaging of PDAC.

### 5.4. Imaging of MUC16

MUC16 has been preclinically evaluated as a target for PET imaging of ovarian tumors, using the mAb B43.13 (oregevomab) or derived fragments conjugated to radionuclides ^64^Cu or ^89^Zr [61,62,63]. In human OVCAR3 tumor-bearing mice, ^89^Zr-B43.13 provided higher TBRs when compared to ^18^F-FDG, which is the gold standard for PET imaging in ovarian cancer [62]. Moreover, the tracer showed uptake in adjacent lymph nodes, which correlated with the lymphatic spread of tumor cells. Since a one-day imaging protocol is more attractive in a clinical setting, the authors attempted using a faster-clearing scFv fragment of B43.13 conjugated to ^18^F in the same in vivo model [63]. Unfortunately, only a modest OVCAR3 tumor uptake was observed. In addition, since mouse B43.13 has been administered to patients and was well tolerated despite human-anti-mouse responses based on serum analysis, clinical application of the tracer seems feasible [62,169].

## 6. Targeting the Glycome: Opportunities and Challenges

**Figure 3:**
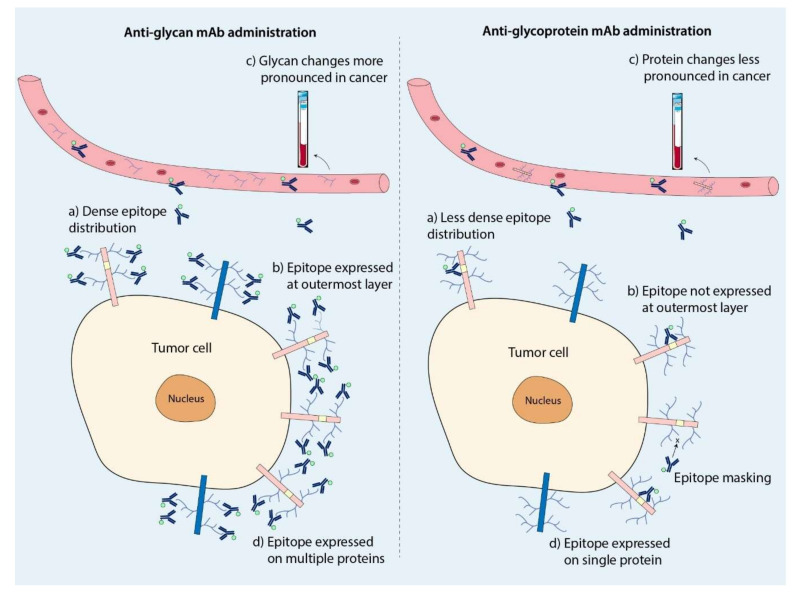
Schematic comparison of glycan-targeting versus traditional (glyco)protein targeting for molecular imaging of tumors. (**a**) Tumor-associated glycans (dark blue branches) are densely packed on multiple proteins (displayed in pink and dark blue) with higher density than binding epitopes on tumor-associated proteins (displayed in yellow). This may result in a denser accumulation of conjugated antibodies, subsequently enhancing tumor signal during imaging. (**b**) Glycans form the outer layer of proteins, making them easily accessible to administered targeting moieties. Noteworthily, glycans may mask binding domains on proteins, challenging specific binding of protein-directed targeting moieties. (**c**) Since aberrantly expressed glycans may be expressed on many glycoproteins (amplified expression), changes in glycoprotein glycosylation are more attractive for use as serum biomarkers than targeting of individual glycoproteins. (**d**) As similar tumor-associated glycan structures are expressed on multiple tumor-associated proteins, glycan-targeting can be more efficient compared to single protein targeting.

Glycan targeting may offer major advantages in relation to protein targeting. Firstly, tumor-associated glycans may be very suitable targets for both therapy and imaging, taking into account their often-low abundance or absence on normal tissues and very dense expression on a wide range of tumors (Figure 3a) [170,171]. Secondly, since glycans are expressed on the outermost layer of the cell surface, they are highly likely to be accessible by administrated targeting vehicles, in contrast to membrane-bound proteins, that may even be masked by glycans (Figure 3b). Thirdly, glycosylation changes may be more pronounced as a response to disease compared to changes in the proteome with atypically-expressed glycans potentially present on many glycoproteins, essentially amplifying their expression [172,173]. These characteristics provide major advantages for imaging of early cancer stages onward, but also for the employment as a serum biomarker for diagnosis, follow-up, monitoring of therapeutic response, or patient stratification, with CA19.9/sLe^a^ as the most illustrative example for monitoring of pancreatic cancer (Figure 3c) [171,174,175]. Most importantly, glycan-directed tracers target multiple tumor-associated proteins simultaneously and provide a broader tumor-targeting strategy than individual protein targeting (Figure 3d). Within this context, especially mucin-type *O-*glycan sTn poses a suitable pan-carcinoma glycotarget, given its high, tumor-specific expression on oncoprotein CD44 as well as MUC1, MUC2, MUC5AC, and MUC6 [84].

Nevertheless, despite over 50 years of glycobiology, glycan-targeting seems still in its infancy. There may be several reasons for this, likely related to difficulties in anti-glycan mAb development [176]. Glycans are not very immunogenic, which results in a major disadvantage. Hybridoma-produced mAbs against glycans are often IgM pentamers that are less optimal, if not unsuitable, for in vivo targeting due to their low affinity and large size, essentially preventing extravasation [106,177,178,179]. Of note, several *N*-glycans are intrinsically expressed by host species used in mAb production, which may explain low glycan immunogenicity. Thus, non-immunoglobin-derived targeting moieties, for which production is less dependent on sufficient immunogenicity, such as aptamers, lectins, and boronic acid derivatives, may represent promising alternatives to mAbs [176]. Alternatively, efforts have been made to improve glycan immunogenicity via various complex immunization protocols, with several successes [83,86]. In addition, the current lack of high-throughput screening methods, which are essential considering the extraordinarily high number of glycan structures, challenges the development of anti-glycan mAbs [176,177,178,179,180]. Since groups of glycans-and particularly *N-*glycans—may be structurally highly related, mAbs are often promiscuous to a certain extent and may, thus, interact with multiple glyco-epitopes, which might be present on normal tissues [181,182]. *N*-glycan targeting is, therefore, regularly overshadowed by the potential of *O-*glycans. However, since novel techniques such as MALDI-TOF-MSI have recently improved *N*-glycan detection and are estimated to increasingly contribute to the identification of novel tumor-specific *N*-glycans, the development of tracers targeting a very specific *N-*glycan structure seems feasible in the near future [183]. In fact, various studies have already indicated that several serum *N-*glycan profiles have extraordinarily high sensitivities and specificities for diagnosis of diverse cancer types [184,185,186]. Lastly, the translation of the results of preclinical glycan-based imaging studies to the human situation is often confounded. Since mice do not express fucosyltransferase-3, a major enzyme involved in Lewis glycan synthesis [187], and their glycome is, in various respects, not directly comparable to humans. This undoubtedly results in overestimation of TBR imaging contrast in studies evaluating these glycans as an imaging target. Therefore, the use of transgenic mice seems inevitable, but the same is true for virtually all protein-directed tracers.

## 7. Conclusions

The search for novel tumor-specific targets for targeted therapy and molecular imaging is an ever-continuing topic of research. Aberrant glycosylation of proteins and lipids as well as overexpression of mucins and proteoglycans is an increasingly relevant feature of cancer, providing tumor cells with unique attributes associated with disease progression. However, the perfect pan-carcinoma target may not exist. Tumor-associated glycans and heavily glycosylated proteins form a panel of targets that deserves extensive attention. As described here, glycan targeting, while remaining challenging, potentially offers major advantages over protein targeting for imaging and therapy. Several promising targeting moieties are currently available, of which some have been already evaluated for imaging and therapeutic purposes. In this review, we summarized the ongoing research within the field of glycan imaging and intended to provide a firm foundation for glycan-based improvement of cancer care in the near future.

## Figures and Tables

**Table 1:** An overview of recent imaging studies evaluating glycans and heavily glycosylated proteins.

Preclinical Studies							
Target	Targeting Moiety	Appl.	Conjugate	Cancer Type	Cell Line	Mouse Model or Phase	Reference
**Thomsen-Friedenreich**	PNA-coated nano-beacon	FI	Coumarin 6	Colon	HCT 116	Subcutaneous	[11]
	TF peptide	PET	^64^Cu-NO2A	Breast	MDA-MB-435	Subcutaneous	[12]
	A38C tetramer	NIRF	AF647	Ovarian Peritoneal Carcinomatosis	IGROV-1	Orthotopic	[13]
**Thomsen-Nouveau**	2154F12A4 mAb	NIRF	Qdot-800	Breast	MCF7	Subcutaneous	[14]
**sialyl-Thomsen-Nouveau**	3E8 scFv	NIRF	IRDye 800	Colon	LS174T	Orthotopic	[15]
	CC49 mAb HuCC49ΔC_H_2 mAb	NIRF	Cy7	Colon	LS174T	Subcutaneous	[16]
	HuCC49ΔC_H_2	PET	^124^I-DOTA	Colon	LS174T	Subcutaneous	[17]
	Pre-target: CC49-HaloTag	SPECT	^111^In-HaloTag ligands	Colon	LS174T	Subcutaneous	[18]
	HuCC49ΔC_H_2	SPECT/γ-scin	^111^In	Colon	LS174T	Subcutaneous	[19]
	CC49 mAb-benzamide-TCO	SPECT/γ-scin	^111^In-Tz	Colon	LS174T	Subcutaneous	[20]
	CC49 mAb-acetamide-TCO	SPECT/γ-scin	^111^In-Tz	Colon	LS174T	Subcutaneous	[21]
**Lewis^a/c/x^**	CH88.2	NIRF	IRDye 800CW	Colon, Pancreas	HT-29, BxPC-3	Subcutaneous	[22]
**sialyl-Lewis^a^**	Anti-CA19-9 mAb	FI	AF488 dye	Pancreas	BxPC-3	Subcutaneous, orthotopic	[23]
	Anti-CA 19-9 mAb	FI	DyLight 650	Pancreas	PDOX	Orthotopic	[24]
	Anti-CA19-9 diabody	PET	^124^I	Pancreas	BxPc-3, Capan-2, MIA PaCa-2	Subcutaneous	[25]
	Anti-CA19-9 cys- diabody	PET	^124^I	Pancreas	BxPC-3	Subcutaneous	[26]
	HuMAb-5B1 mAb	PET	^89^Zr-DFO123456	Bladder	HT 1197	Subcutaneous	[27]
	Pre-targeted: 5B1 mAb	PET	^89^Zr	Pancreas	Capan-2	Subcutaneous, orthotopic	[28]
	5B1 mAb-TCO	PET	^64^Cu-NOTA-PEG7-Tz	Pancreas	BxPC-3, Capan-2	Subcutaneous, orthotopic	[29]
	PEGPH20 and HuMab-5B1 mAb	PET	^89^Zr-DFO	Pancreas	BxPC3-HAS3	Subcutaneous	[30]
	5B1 mAb	PET/NIRF	^89^Zr-DFO FL dye	Pancreas	BxPC-3, MIA PaCa-2, Suit-2	Subcutaneous, orthotopic	[31]
**sialyl-Lewis^x^**	sLeX-carrying liposomes	NIRF	Cy5.5	-	Ehrlich Ascites tumor	Subcutaneous	[32]
**Lewis^y^**	hu3S193 mAb	PET SPECT/γ-scin	^111^In, ^86^Y	Colon	HCT-15	Subcutaneous	[33]
	hu3S193 diabody F(ab’)_2_	SPECT/γ-scin	^111^In-CHX-A”-DTPA	Breast	MCF-7	Subcutaneous	[34]
**GD2**	ch14.18-CHO	NIRF	IRDye 800CW	Neuroblastoma	KCNR, patient-derived	Orthotopic	[35]
	hu14.18K322A mAb	PET	^64^Cu-*p*-NH2-Bn-DOTA	Neuroblastoma, melanoma	M21, PC-3.	Subcutaneous	[36]
	hu14.18K322A mAb	PET	^64^Cu-Bn-NOTA	Osteosarcoma	SJOS072	Subcutaneous	[37]
**Syndecan-1**	Recombinant syndecan-1	MSOT	CF750 succinyl ester	Pancreas	S2VP10	Orthotopic	[38]
	9E7.4 mAb	PET	^64^Cu-TE2A	Multiple myeloma	5T33	Subcutaneous, orthotopic	[39]
**Glypican-1**	Glypican-1 mAb	FI/MRI	Gd-Au-nanoclusters	Pancreas	COLO-357	Subcutaneous	[40]
**Glypican-3**	Pretarget: L5 peptide	MRI	SA-PEG-USPIO	HCC	HepG2	Subcutaneous	[41]
	TJ12P1 peptide	NIRF	Cy5.5	HCC, Prostate	HepG2, PC3	Subcutaneous	[42]
	αGPC3 mAb	PET	^89^Zr	HCC	HepG2	Orthotopic	[43]
	αGPC3 F(ab’)_2_	PET	^89^Zr	HCC	HepG2	Orthotopic	[44]
**MUC1**	EPPT peptide	MRI/NIRF	Magnetic NP-Cy5.5	Colon	MC38 MUC1	Orthotopic	[45]
	EPPT peptide	MRI/NIRF	Magnetic NP-Cy5.5	Breast	Spontaneous	Orthotopic	[46]
	EPPT peptide	MRI/NIRF	Magnetic NP-Cy5.5	Pancreas	Spontaneous	Orthotopic	[47]
	EPPT peptide	MRI/NIRF	Magnetic NP-Cy5.5	Colon	LS174T	Subcutaneous	[48]
	CT2 mAb	NIRF	DyLight 650	Pancreas	Panc-1, BxPC-3	Subcutaneous, orthotopic	[49]
	hMUC1 mAb	NIRF	DyLight 755	Pancreas	Capan-2	Subcutaneous	[50]
	CD227 mAb	NIRF	Fluorescein-Cy5.5	Ovary	OVCAR3	Subcutaneous	[51]
	MUC1 aptamer	NIRF	MPA-PEG	Breast, liver	MCF-7, HepG2	Subcutaneous	[52]
	TAB 004 mAb	NIRF	ICG	Breast	PyMT, MMT, spontaneous	Orthotopic	[53]
	GGSK-1/30	PET/MRI	^89^Zr	Breast	PyMTxhuMUC1	Subcutaneous	[54]
	PR81 mAb	PET/SPECT	^64^Cu-DOTA	Breast	MCF-7	Subcutaneous	[55]
	PR81 mAb	SPECT/γ-scin	^99m^Tc	Breast	Spontaneous	Orthotopic	[56]
**MUC1/MUC5AC**	Pretarget: TF10 bispecific mAb	γ-scin	^125^I-IMP-288	Pancreas	Capan-1	Subcutaneous	[57]
	bsPAM4 F(ab’)_2_	γ-scin	^125^I	Pancreas	Capan-1	Subcutaneous	[58]
	Pretarget: bsPAM4 F(ab’)_2_	γ-scin	^111^In-IMP-156 ^99m^Tc-IMP-192	Pancreas	Capan-1	Subcutaneous	[58]
**MUC5AC**	60C peptide	MRI	USPIO	Colon	HT-29, HCT 116	Subcutaneous	[59]
**MUC16**	AR9.6 mAb	NIRF	IRDye 800CW	Pancreas	COLO 357, T3M4	Subcutaneous, orthotopic	[60]
	B43.13 mAb B43.13 scFv	PET	^64^Cu	Ovary	OVCAR3, SKOV3	Subcutaneous	[61]
	B43.13 mAb	PET	^89^Zr	Ovary	OVCAR3, SKOV3	Subcutaneous	[62]
	B43.13 mAb B43.13 scFv	PET	^18^F (FBz)	Ovary	OVCAR3, SKOV3	Subcutaneous	[63]
**Clinical Studies**							
**sialyl-Thomsen-Nouveau**	B72.3 mAb	RIGS	^125^I	Colon, rectum	-	Phase 1/2	[64]
	CC49 mAb	RIGS	^125^I	Colon, rectum	-	Phase 1	[65]
	CC49 mAb	RIGS	^125^I	Colon, rectum	-	Phase 1	[66]
	HuCC49ΔC_H_2 mAb	RIGS	^125^I	Colon, rectum	-	Phase 1	[67]
	HuCC49ΔC_H_2 mAb	RIGS	^125^I	Colon, rectum	-	Phase 1	[68]
**sialyl-Lewis^a^**	HuMAb-5B1 mAb	PET	^89^Zr-DFO	Pancreas	-	Phase 1	[69]
	B3 mAb	SPECT	^111^In	Various	-	Phase 1	[70]
**Lewis^y^**	hu3S193 mAb	SPECT/γ-scin	^111^In	Lung	-	Phase 1	[71]
**GD2**	ch14.18 mAb	γ-scin	^99m^Tc	Neuroblastoma	-	Phase 1	[72]
	3F8 mAb	γ-scin	^131^I	Neuroblastoma	-	Phase 1	[73]
	3F8 mAb	PET	^124^I	Neuroblastoma	-	Case report	[74]
**MUC1**	C595 mAb	γ-scin	^111^In	Bladder	-	Phase 1	[75]
	C595 mAb	γ-scin	^99m^Tc	Bladder	-	Phase 1	[76]
**MUC1/MUC5AC**	hPAM4 mAb	γ-scin	^111^In	Pancreas	-	Phase 1	[77]

Abbreviations: Appl.: application, CHX-A”-DTPA: C-functionalized Trans-cyclohexyl Diethylenetriaminepentaacetic acid, γ-scin: Gamma Scintigraphy, DFO: Desferrioxamine, DOTA: 1, 4, 7, 10-Tetraazacyclododecane-1, 4, 7, 10-Tetra-Acetic Acid, GSG: Gly-Ser-Gly, FI: Fluorescence Imaging, HCC: Hepatocellular Carcinoma, ICG: Indocyanine Green, PEG: Polyethylene Glycol, MAb: Monoclonal Antibody, MSOT: Multispectral Optoacoustic Imaging, MRI: Magnetic Resonance Imaging, NIRF: Near-Infrared Fluorescence, NP: Nanoparticle NOTA: 1, 4, 7-Triazacyclononane-1, 4, 7-Triacetic Acid, NO2A: 1, 4, 7-Triazacyclononane-1, 4-Diacetate, PDOX: Patient-Derived Orthotopic Xenograft, PNA: Arachis Hypogaea Agglutinin, RIGS: radioimmunoguided surgery, SA: Streptavidin, TCO: Trans-Cyclooctene, Tz: Tetrazine, USPIO: Ultrasmall Superparamagnetic Iron Oxide.
